# Molecular Characteristics and Factors Associated with Antimicrobial Resistance in *Campylobacter* Isolates from Small-Scale Chicken Farms in Southern Thailand

**DOI:** 10.3390/antibiotics15080756

**Published:** 2026-08-05

**Authors:** Nguyen Thi My Nhan, Doan Hoang Phu, Phitchayapak Wintachai, Thotsapol Thomrongsuwannakij

**Affiliations:** 1 Akkhraratchakumari Veterinary College, Walailak University, Nakhon Si Thammarat 80160, Thailand; nhan.nguyenthimy@hcmuaf.edu.vn; 2 Doctoral Program in Health Sciences, College of Graduate Studies, Walailak University, Nakhon Si Thammarat 80160, Thailand; 3 Faculty of Animal Science and Veterinary Medicine, Nong Lam University, Ho Chi Minh City 71309, Vietnam; dhoangphu@hcmuaf.edu.vn; 4 Department of Agriculture, Nong Lam University, Gia Lai Campus, Pleiku 61125, Vietnam; 5 School of Science, Walailak University, Nakhon Si Thammarat 80160, Thailand; sanwinta@gmail.com; 6 Centre for One Health, Walailak University, Nakhon Si Thammarat 80160, Thailand

**Keywords:** *Campylobacter jejuni*, Campylobacter coli, antimicrobial resistance, virulence genes, small-scale farms, southern Thailand

## Abstract

**Background/Objectives:** *Campylobacter* spp. are leading causes of foodborne gastroenteritis, and small-scale poultry farms are important reservoirs of antimicrobial resistance (AMR). This study investigated the prevalence of *Campylobacter* spp., AMR profiles, virulence genes, and associated risk factors of *Campylobacter* isolates in small-scale chicken farms in southern Thailand. **Methods:** Cloacal swab samples were collected from 10 randomly selected birds at three time points across 12 farms. Isolates were identified by multiplex PCR. Antimicrobial susceptibility was determined by agar dilution. Resistance-associated genes (*gyrA*, 23S rRNA A2075G/A2074C, *cmeABC*, *tet(O)*) and virulence genes (*flaA*, *flhA*, *cadF*, *cdtABC*) were detected using PCR. Risk factors were analyzed using logistic regression. **Results:** Of 358 cloacal samples, 151 were positive for *Campylobacter* spp. (overall weighted prevalence: 53.56%). A total of 153 isolates were recovered from positive samples of 142 *C. jejuni* (weighted prevalence: 50.04%) and positive samples of 11 *C. coli* (crude prevalence: 3.07%), as two samples contained mixed infections of both species. For *C. jejuni*, weighted resistance was high for CIP (91.44%), NAL (90.10%), and TET (64.22%), while erythromycin resistance was absent (0.00%), and MDR was low (0.70%). For *C. coli*, resistance reported as crude proportions was 81.82% for CIP, 72.73% for NAL, 45.45% for TET, 0.00% for ERY, and 9.09% for MDR. The *gyrA* Thr-86-Ile mutation was associated with quinolone and fluoroquinolone resistance, and the A2074C mutation with tylosin resistance. The *cmeABC* operon was found in all isolates. The *cdt* virulence cluster was present in all *C. jejuni*, but largely absent in *C. coli*. Multivariable modelling showed that prevalence was associated with larger flock size, inadequate manure management, and overall antimicrobial use, whereas AMR was associated with farm location, owner age, and specifically tetracycline use. **Conclusions:** High prevalence and molecular diversity of *Campylobacter* highlight the need for improved antimicrobial stewardship and biosecurity in small-scale poultry systems.

## 1. Introduction

*Campylobacter* spp. are among the leading causes of bacterial gastroenteritis worldwide and contribute substantially to the global burden of foodborne disease [1,2,3]. Poultry, particularly chickens, is recognized as the primary reservoir for human *Campylobacter* infections, with transmission occurring mainly through the consumption or handling of contaminated poultry products [4,5]. In recent decades, the rising prevalence of antimicrobial-resistant *Campylobacter* has emerged as a major public health concern. Fluoroquinolone-resistant *Campylobacter* strains are increasingly recognized as high-priority pathogens requiring urgent monitoring and control through antimicrobial resistance (AMR) surveillance systems [3,6,7,8]. This trend raises concerns regarding clinical treatment efficacy in humans and reflects the risk of persistence and dissemination of resistant *Campylobacter* within food production systems.

At the molecular level, antimicrobial resistance in *Campylobacter* is driven primarily by chromosomal mutations and efflux-mediated mechanisms. Point mutations in the quinolone resistance-determining region (QRDR) of the *gyrA* gene, particularly the threonine-86-isoleucine substitution (Thr-86-Ile), are strongly associated with quinolone and fluoroquinolone resistance [9]. Macrolide resistance is linked to mutations in domain V of the 23S rRNA gene, notably A2075G and A2074C, with phenotypic expression influenced by the extent of these mutations and efflux activity [10]. The CmeABC efflux pump reduces susceptibility across multiple antimicrobial classes and acts as a resistance modulator, whereas *tet(O)* mediates tetracycline resistance through ribosomal protection [11,12,13]. In addition to resistance determinants, several virulence-associated genes, including *flaA*, *cadF*, and the cytolethal distending toxin genes (*cdtA*, *cdtB*, *cdtC*), are essential for motility, adhesion, colonization, and host cell damage [14,15]. The co-occurrence of AMR and virulence determinants has been reported in poultry-associated *Campylobacter* isolates and may indicate strains of greater public health concern [16,17]. This phenomenon is particularly pertinent to small-scale poultry systems, where close human–animal contact occurs [18]. Importantly, the extent to which antimicrobial usage (AMU) patterns and molecular resistance determinants jointly influence the phenotypic expression of AMR in small-scale poultry systems poses more significant challenges to understanding this issue [19,20]. Conditions in these systems, including inadequate biosecurity, unregulated antimicrobial use, and close human–animal contact, may promote the emergence and dissemination of antimicrobial-resistant *Campylobacter* across animal, human, and environmental interfaces [21,22].

In Thailand, poultry production plays a vital role in both domestic consumption and the agricultural economy. Alongside large-scale commercial operations, small-scale and backyard chicken farming systems are widely practiced, particularly in rural and peri-urban areas. These systems often operate with limited veterinary oversight and antimicrobial stewardship, increasing the risk of inappropriate AMU [20]. Although Campylobacter and AMR in poultry have been reported in Thailand, evidence focusing specifically on small-scale farms in southern Thailand remains limited, particularly studies that simultaneously integrate phenotypic resistance profiles, molecular determinants (including AMR and virulence genes), antimicrobial use practices, and farm-level epidemiological factors such as biosecurity [23,24]. This lack of integrated evidence combining molecular characterization with farm-level risk factor assessment constrains the development of targeted intervention strategies tailored to small-scale production systems. Furthermore, this gap is particularly critical given the heterogeneous AMU practices in small-scale poultry production, which may directly influence the emergence, maintenance, and dissemination of antimicrobial-resistant *Campylobacter*. To address this gap, this study applied an integrated framework combining phenotypic, genotypic (AMR and virulence profiling), and farm-level epidemiological data within a repeated cross-sectional design to provide a comprehensive perspective on resistant *Campylobacter* spp. in small-scale poultry systems in Thailand.

Therefore, this study aimed to investigate the molecular and epidemiological determinants of AMR in *Campylobacter* spp. isolated from small-scale chicken farms in Southern Thailand. Specifically, the study aimed to: (i) investigate the prevalence of *Campylobacter* spp. across different stages of the small-scale chicken production cycle; (ii) characterize phenotypic and genotypic AMR profiles and their temporal dynamics among *Campylobacter* isolates; (iii) determine the distribution of resistance-associated genes and virulence determinants; (iv) identify the farm-level risk factors, including AMU patterns, associated with the occurrence and AMR status of *Campylobacter* spp. By integrating phenotypic, genotypic, and farm-level data within a longitudinal framework, this study provides a comprehensive perspective on the emergence and maintenance of AMR of *Campylobacter* spp. in small-scale poultry systems in Thailand.

## 2. Results

### 2.1. Prevalence and Species Distribution of Campylobacter spp.

**Figure 1:**
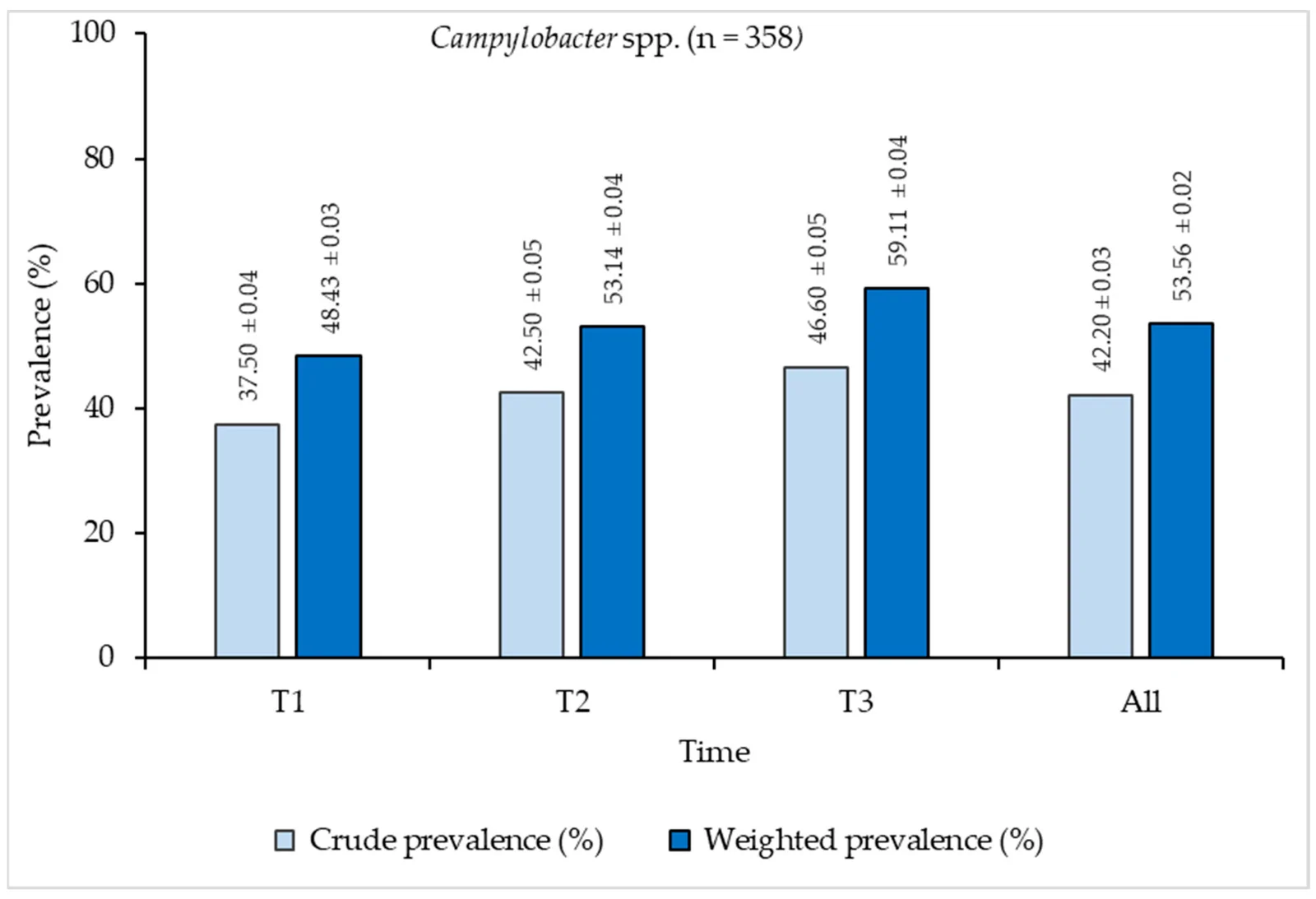
Crude and weighted prevalence of *Campylobacter* spp. across production stages.

A total of 358 cloacal swab samples were collected from 12 small-scale chicken farms across three sampling time points (Time 1: n = 120; Time 2: n = 120; Time 3: n = 118). The reduced sample size at Time 3 (n = 118) resulted from partial flock sale before the scheduled sampling visit, leaving fewer than 10 birds available on one farm. Of these, 151 samples (42.2%) tested positive for *Campylobacter* spp. After adjustment for farm-level sampling weights, the weighted prevalence was 53.56% (SE ± 0.02). Weighted prevalence increased progressively across time points, from 48.43% (SE ± 0.03) at Time 1 to 53.14% (SE ± 0.04) at Time 2 and 59.11% (SE ± 0.04) at Time 3 (Figure 1).

**Figure 2:**
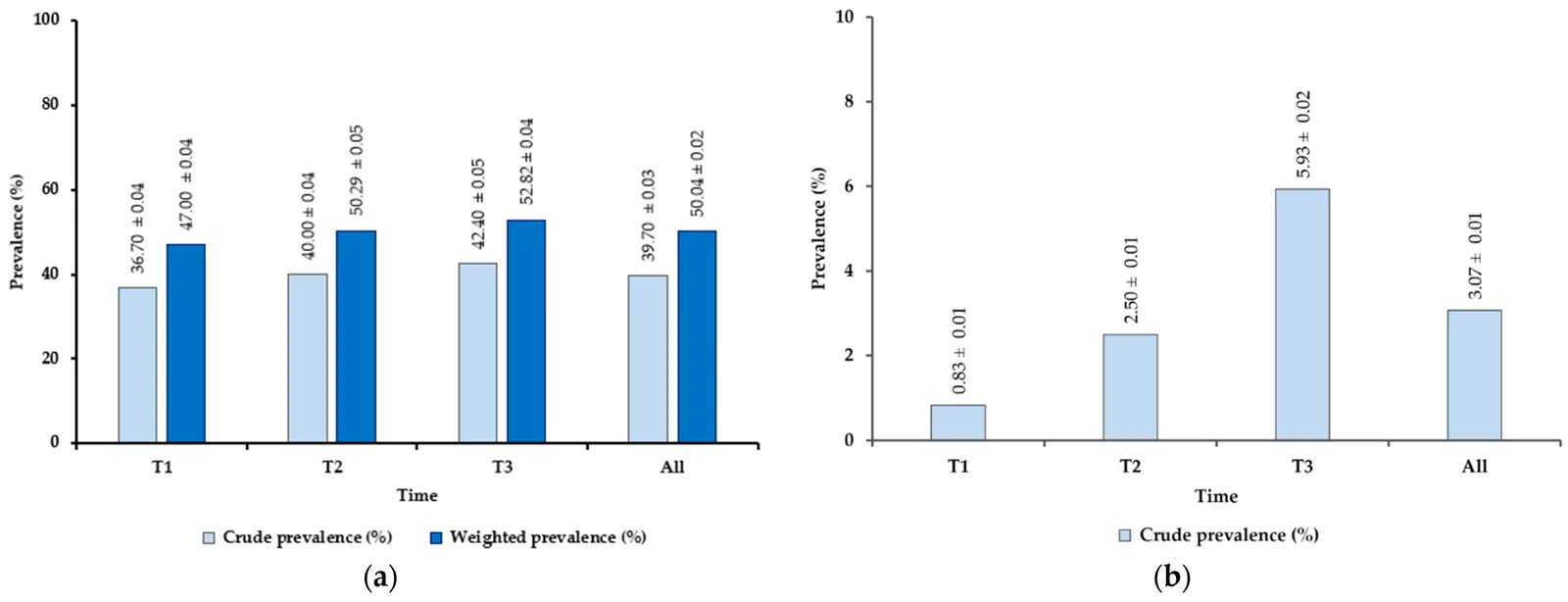
Prevalence of *C. jejuni* (**a**) and *C. coli* (**b**) across production stages.

Out of 151 positive samples, there were 142 *C. jejuni* and 11 *C. coli* isolates, with two samples co-infected with both species simultaneously, resulting in 153 isolates in total. The weighted prevalence of *C. jejuni* was 50.04% (SE ± 0.02) overall, increasing from 47.00% at Time 1 to 50.29% at Time 2 and 52.82% at Time 3 (Figure 2a). The crude prevalence of *C. coli* was 3.07% (11/358) overall, with values of 0.83% (1/120) at Time 1, 2.50% (3/120) at Time 2, and 5.93% (7/118) at Time 3 (Figure 2b). Due to the small isolate recovery for *C. coli* (n = 11), resistance for *C. coli* was reported as crude proportions (n/N, %) without survey weighting.

### 2.2. Phenotypic Antimicrobial Resistance

The MIC distributions and resistance classification for six antimicrobial agents were determined for 142 *C. jejuni* and 11 *C. coli* isolates. Because of the small *C. coli* denominator (n = 11), only crude prevalence is reported for that species. MIC distributions for both species, with crude prevalence, are provided in Table 1. Resistance to quinolones and fluoroquinolones was the most prominent finding across both species, with very high resistance levels observed for CIP; the weighted prevalence of resistance in *C. jejuni* was 91.44%, and the crude prevalence in *C. coli* was 81.82% (9/11). For NAL and ENR, the weighted prevalence in *C. jejuni* was 90.10% and 73.73%, respectively, while the crude prevalence in *C. coli* was 72.73% (8/11) for both agents. Macrolide resistance was entirely absent for ERY in both species across all time points, with 100% of *C. jejuni* (142/142) and *C. coli* (11/11) isolates classified as susceptible. For TYL, resistance was rare in *C. jejuni* (weighted prevalence 0.95%) and reached a crude 27.27% (3/11) in *C. coli*. Tetracycline resistance was common, with a weighted prevalence of 64.22% in *C. jejuni* and a crude prevalence of 45.45% (5/11) in *C. coli*.

**Figure 3:**
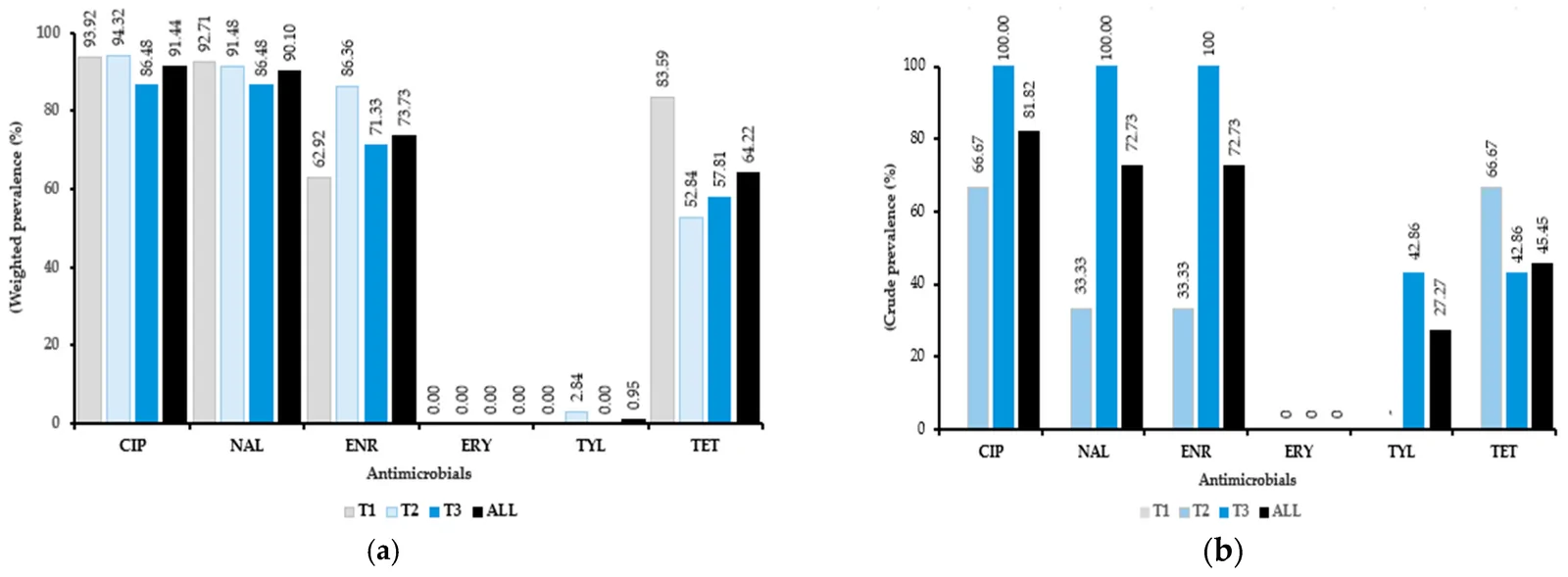
AMR patterns in *Campylobacter* isolates across production stages (T1–T3) and overall. Weighted prevalence of resistance in *C. jejuni* (**a**). Crude prevalence of resistance in *C. coli* (**b**). Abbreviations: CIP: ciprofloxacin; NAL: nalidixic acid; ENR: enrofloxacin; ERY: erythromycin; TYL: tylosin; TET: tetracycline.

Temporal AMR levels of *C. jejuni* and *C. coli* are shown in Figure 3. In *C. jejuni*, there was a high prevalence of CIP resistance at Time 1–3, 93.92%, 94.32%, and 86.48%, respectively. Similarly, NAL resistance levels were 92.71%, 91.48%, and 86.48% at 3 different time points of sample selection. ENR resistance varied more (62.92%, 86.36%, 71.33%). ERY resistance was 0.00% at all time points. TYL resistance occurred only at Time 2 (2.84%). TET resistance was highest at Time 1 (83.59%), fell at Time 2 (52.84%), and rose partially at Time 3 (57.81%). Descriptively, in *C. coli*, the single Time 1 isolate was pan-susceptible. At Time 2 (n = 3), crude resistance was 66.67% for CIP and 33.33% for NAL and ENR; at Time 3 (n = 7), all three reached 100%. TYL resistance in *C. coli* appeared only at Time 3 (42.86%); TET resistance was 66.67% at Time 2 and 42.86% at Time 3.

The distribution of AMR profiles and multidrug resistance (MDR) is summarized in Table 2. MDR was detected in 2 of 153 isolates (crude 1.31%): one *C. jejuni* (crude 0.70%; weighted 0.95%) and one *C. coli* (crude 9.09%). In *C. jejuni* (n = 142), nine AMR profiles were identified. The predominant profile was two-class resistance combining quinolones and fluoroquinolones and tetracycline (CIP-ENR-NAL-TET; 45.07%, 64/142), followed by single-class quinolones and fluoroquinolones resistance (CIP-ENR-NAL; 26.76%, 38/142), CIP-NAL-TET (13.38%, 19/142), and CIP-NAL (4.93%, 7/142). Profiles of CIP-TET (1.41%, 2/142), ENR-NAL-TET (0.70%, 1/142), and TET alone (0.70%, 1/142) were infrequent. Seven isolates (4.93%) were pan-susceptible. Descriptively, in *C. coli* (n = 11), six profiles were identified, most commonly CIP-ENR-NAL-TET (27.27%, 3/11) and CIP-ENR-NAL-TYL (18.18%, 2/11), followed by CIP-ENR-NAL (18.18%, 2/11), CIP alone (9.09%, 1/11), and TET alone (9.09%, 1/11); one isolate (9.09%) was pan-susceptible.

### 2.3. Genotypic AMR Profiles and Determinants

**Figure 4:**
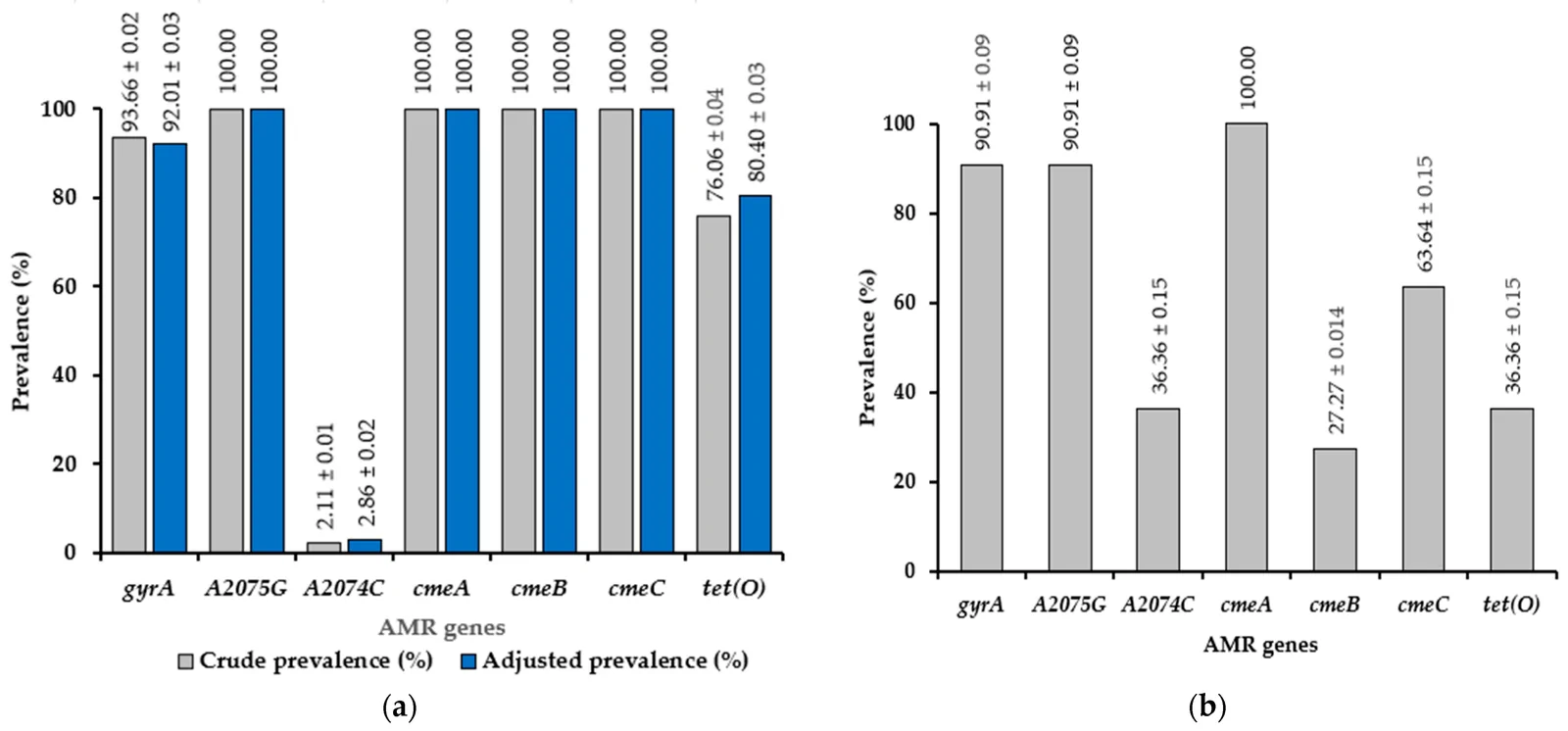
Distribution of AMR genes in *C. jejuni* (**a**) and *C. coli* (**b**) isolates.

Resistance gene distributions are shown in Figure 4. In *C. jejuni* (n = 142), the *gyrA* Thr-86-Ile substitution was detected in 92.01% of isolates, the 23S rRNA A2075G mutation in 100%, and A2074C in 2.86%. All three *cmeABC* efflux pump genes were present in 100% of isolates (*cmeA*, *cmeB*, *cmeC*; each 100%). The *tet(O)* gene was detected in 80.40% of *C. jejuni* isolates. Descriptively, in *C. coli* (n = 11), the prevalence of the *gyrA* mutation was 90.91% and A2075G was 90.91%, while A2074C was 36.36%. Genes *cmeA* and *cmeC* were detected in 100% and 63.64% of isolates, respectively, whereas *cmeB* was detected in 27.27%. The *tet(O)* gene was present in 36.36% of *C. coli* isolates.

Across time points (Table 3), the *gyrA* mutation in *C. jejuni* remained high (Time 1, 92.70 ± 0.04%; Time 2, 94.32 ± 0.04%; Time 3, 89.18 ± 0.05%). A2075G was detected in 100% of *C. jejuni* at all time points, whereas A2074C occurred at low frequency (Time 1, 3.04 ± 0.03%; Time 2, 5.68 ± 0.04%; Time 3, 0.00%). All three *cmeABC* genes were present in 100% of *C. jejuni* at every time point. *tet(O)* ranged from 78.12 ± 5.57% (Time 1) to 79.55 ± 0.06% (Time 2) and 83.23 ± 0.04% (Time 3). In *C. coli*, the A2074C mutation was absent at Time 1 and Time 2 but reached 57.14 ± 0.20% at Time 3, while *cmeB* and *cmeC* detection was lower and more descriptively variable across time points.

### 2.4. Agreement Between Phenotypic and Genotypic Resistance

The agreement between phenotypic resistance and genotypic determinants across all antimicrobial classes is summarized in Table 4.

For quinolones and fluoroquinolones, the *gyrA* Thr-86-Ile mutation showed substantial agreement with CIP resistance (κ = 0.744, *p* < 0.001) and NAL resistance (κ = 0.639, *p* < 0.001). Agreement with ENR was fair (κ = 0.241, *p* < 0.001); 28 *C. jejuni* isolates with ENR MIC = 2 µg/mL were classified as intermediate under CLSI breakpoints (S ≤ 1; I = 2; R ≥ 4 µg/mL) yet carried the *gyrA* mutation. The *cmeABC* genes showed limited to no agreement with quinolone and fluoroquinolone resistance: *cmeA* was universal and not computable, while kappa values of *cmeB* and *cmeC* ranged from −0.004 to 0.305.

For macrolides, all 153 isolates were erythromycin-susceptible (no resistant isolates); kappa was therefore not computable for A2075G, A2074C, or the cmeABC components against erythromycin. A2075G was detected in 152 isolates and A2074C in 7, yet none were erythromycin-resistant by MIC. For tylosin (4 resistant isolates), agreement was substantial with A2074C (κ = 0.718, *p* < 0.001): all four tylosin-resistant isolates carried A2074C, and three further A2074C-positive isolates were susceptible (7 A2074C-positive in total). Agreement with A2075G was negligible (κ = 0.000, *p* = 0.869): all four resistant isolates also carried A2075G, but it was also widely present in 148 susceptible isolates (152 A2075G-positive in total), leaving no agreement beyond chance.

And, for tetracycline, *tet(O)* showed moderate agreement (κ = 0.503, *p* < 0.001), with 86 concordant resistant isolates, 8 phenotype-positive/genotype-negative isolates, and 26 phenotype-negative/genotype-positive isolates. *cmeABC* genes showed slight or negligible agreement with tetracycline resistance (κ = 0.015–0.030).

### 2.5. Virulence Gene Profiles

**Figure A1:**
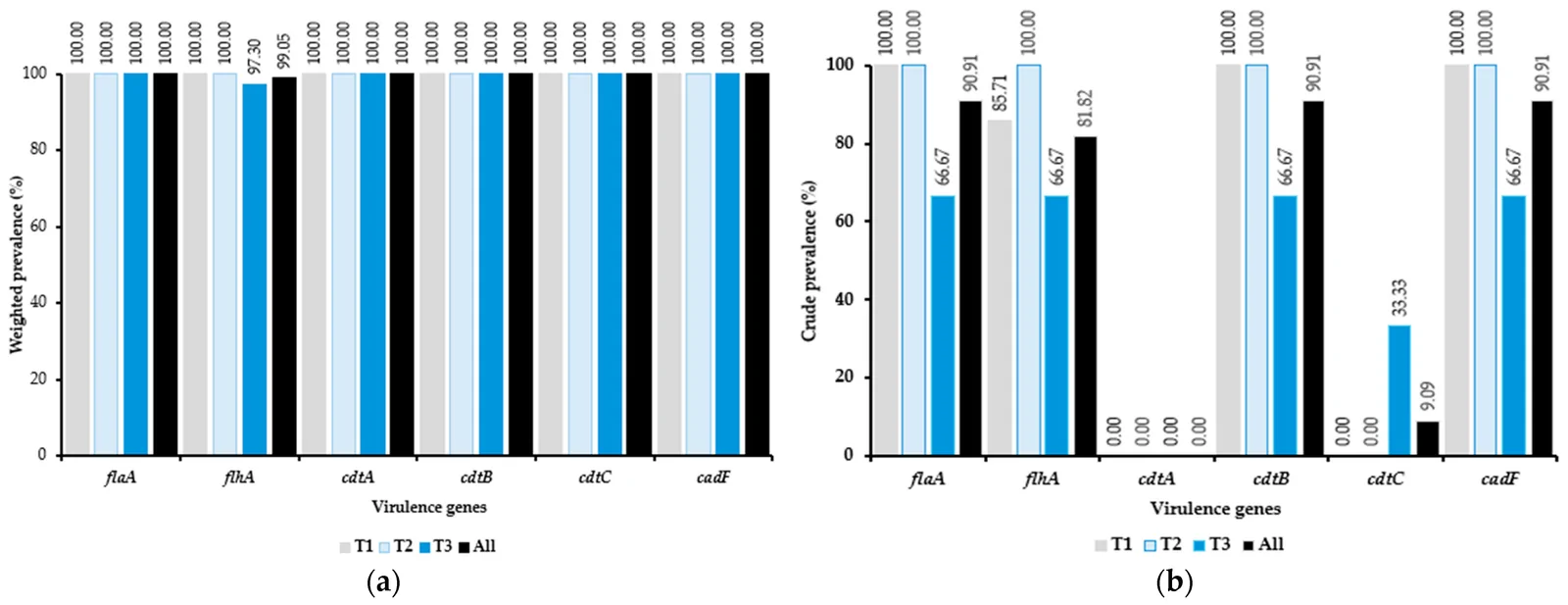
Distribution of virulence genes in *C. jejuni* (**a**) and *C. coli* (**b**) isolates across production stages.

Virulence gene distributions are shown in Figure A1. In *C. jejuni*, genes *flaA*, *cdtA*, *cdtB*, *cdtC*, and *cadF* each reached 100% weighted prevalence at all time points and overall, while gene *flhA* was 100% at Time 1 and Time 2 and 97.30% at Time 3 (overall weighted 99.05%). In *C. coli*, virulence gene distribution was descriptively variable (based on crude prevalence). Genes *flaA*, *cdtB*, and *cadF* each had an overall prevalence of 90.91% (100% at Time 1 and Time 2; 66.67% at Time 3). Gene *flhA* was 81.82% overall (85.71% at Time 1, 100% at Time 2, 66.67% at Time 3). The *cdt* cluster diverged markedly between species: *cdtA* was absent in all *C. coli* isolates (0%), *cdtB* was present in 90.91%, and *cdtC* was detected in only one Time 3 isolate (overall 9.09%).

### 2.6. Farm-Level Risk Factors for Campylobacter Occurrence and CIP–TET Co-Resistance

Univariable and multivariable logistic regression results for *Campylobacter* positivity are shown in Table 5. In univariable analysis, factors significantly associated with positivity included Pho Thong district, owner age > 50 years, secondary education, flock size > 60 birds, pen area > 300 m^2^, rice husk litter, wooden litter, heating system, cattle presence, inadequate manure management, absence of biosecurity washing, feeder not cleaned, and tetracycline use (all *p* ≤ 0.034). In the final multivariable model, three factors remained independently associated with *Campylobacter* positivity: flock size > 60 birds (aOR = 3.15, 95% CI 1.74–5.85, *p* < 0.001), inadequate manure management (aOR = 9.65, 95% CI 4.84–20.92, *p* < 0.001), and antimicrobial use (aOR = 3.85, 95% CI 1.36–12.13, *p* = 0.015). This final model demonstrated optimal goodness-of-fit with the lowest AIC value (AIC = 358.20).

For the second model, CIP–TET co-resistance was selected as the outcome variable because it represented the predominant antimicrobial resistance phenotype among isolates (91/151, 60.3%), reflecting the high selection pressure from these commonly used antimicrobial classes (Table 6). In univariable analysis, factors significantly associated with co-resistance included sampling time in April 2025, sampling time in June 2025, owner age > 50 years, secondary education, farming experience of 6 to 10 years, farming experience > 10 years, rice husk litter, heating in pen, presence of other animals on farm, cattle presence, and concrete flooring (all *p* ≤ 0.05). In the final multivariable model, location (Pho Thong), owner age > 50 years, and tetracycline use were retained. Hua Taphan location (aOR = 9.00, *p* < 0.001), owner age ≤ 50 years (aOR = 13.34, *p* < 0.001), and tetracycline use (aOR = 5.67, *p* = 0.002) were retained. This final model achieved the best model fit with an AIC value of 176.85.

## 3. Discussion

Through a longitudinal survey integrating prevalence, phenotypic susceptibility testing, resistance and virulence gene profiling, genotype–phenotype agreement analysis, and farm-level risk-factor modelling, this study addresses the gap related to limited biosecurity and unregulated AMU, which favour both colonization and persistence of resistant *Campylobacter* in small-scale chicken farms in southern Thailand. Four key findings are highlighted: (i) *Campylobacter* colonization was common and increased across the production cycle, with *C. jejuni* predominating; (ii) quinolone and fluoroquinolone resistance was high, tetracycline was moderate, while erythromycin resistance was effectively absent; and genotype–phenotype agreement varied by antimicrobials; (iii) virulence genes were highly consistent in *C. jejuni*, while *C. coli* exhibited more variable profiles; and (iv) factors associated with *Campylobacter* prevalence included larger flock size, inadequate manure management, and overall antimicrobial use, whereas AMR was associated with farm location, owner age, and specifically tetracycline use.

The prevalence of *Campylobacter* spp. (53.56%) indicates that small-scale chicken farms in southern Thailand may represent an important reservoir of *Campylobacter* with potential relevance to human exposure. The prevalence of *C. jejuni* (50.04%) is broadly consistent with the 43.6% pooled estimate reported in a recent regional meta-analysis of Thai chickens [25]. The predominance of *C. jejuni* over *C. coli* (50.04% versus 3.07%) aligns with the species distribution reported in Thai commercial broiler and Thai retail chicken systems [25,26,27]. Importantly, detection of mixed *C. jejuni* and *C. coli* colonization in two birds suggests that small-scale flocks can carry both species concurrently, consistent with the heterogeneous exposure conditions typical of smallholder farms in the region [28,29]. Furthermore, the increasing prevalence over the production cycle (Time 1, 48.43%; Time 2, 53.14%; Time 3, 59.11%) may reflect cumulative environmental exposure in low-biosecurity systems [28,30]. Although sampling time was significantly associated with co-resistance in univariable analysis, it was not retained in the final multivariable model after adjusting for farm-level predictors. Thus, observed fluctuations across visits (Figure 3) represent descriptive temporal patterns rather than statistically confirmed AMR trends, underscoring the need for extended longitudinal monitoring.

High fluoroquinolone resistance in *C. jejuni* is consistent with persistently high resistance reported in poultry-associated *Campylobacter* in Thailand, with the ciprofloxacin estimate (91.44%) slightly exceeding the nationwide pooled prevalence (71.6% to 88.7%) [25]. This difference may reflect the focus on native chickens in the present study, compared with mixed production systems (both broilers and native chickens) in previous analyses, and suggests that lower biosecurity levels and unregulated AMU in small-scale poultry systems may contribute to higher AMR prevalence [18,31]. The lower ENR estimate relative to CIP, despite shared *gyrA*-associated quinolone resistance, likely reflects a higher proportion of isolates falling within the intermediate range, rather than a distinct resistance mechanism, resulting in fewer isolates being classified as resistant compared with CIP and NAL. In contrast, no ERY resistance was observed in *C. jejuni*, consistent with previous reports indicating very low macrolide resistance in *C. jejuni* (1.8%), and higher in *C. coli* (37.7%) [25]. The preserved susceptibility is clinically important, as macrolides remain the treatment of choice for severe *Campylobacter* infections in the context of widespread quinolone and fluoroquinolone resistance [3,10]. TET resistance in *C. jejuni* is consistent with previous poultry-associated *Campylobacter* data from southern Thailand [25]; and is supported by multivariable analysis showing an association between tetracycline use and AMR. Given the high baseline prevalence of fluoroquinolone resistance, this association was largely driven by tetracycline resistance. Furthermore, because AMU was recorded qualitatively, exposure intensity and direct causal relationships between overall AMU and resistance could not be established. Although resistance levels for tetracycline remained lower than those observed for quinolones, this pattern likely aligns with reported qualitative differences in farm-level AMU and environmental exposure among study flocks [30].

The *gyrA* Thr-86-Ile substitution showed substantial agreement with CIP and NAL resistance, supporting its role as the main marker of fluoroquinolone resistance in *C. jejuni* [9,32,33,34]. The substantial agreement observed for *gyrA* and the moderate agreement of *tet(O)* with phenotypic tetracycline resistance align closely with earlier reports [34]. The presence of tetracycline-resistant, *tet(O)*-negative isolates suggests additional mechanisms, such as efflux effects or undetected resistance determinants [35]. Conversely, the poor genotype–phenotype agreement for erythromycin contrasts with previous studies where 23S rRNA mutations strongly predicted macrolide resistance [10,11,36]. Here, widespread A2075G detection without observed ERY resistance indicates that point mutations alone may not guarantee clinical resistance. This observed pattern could be influenced by several factors, including low-level expression and point mutations present in only a single 23S rRNA operon copy or variations in gene expression. In contrast, all four TYL-resistant isolates carried A2074C (kappa = 0.718), although this finding remains exploratory due to the small number of resistant isolates. Overall, these observations underscore how target gene mutations alone may not fully account for phenotypic expression.

Our resistance profile is consistent with reports from other poultry production systems and poultry species. High quinolone, fluoroquinolone, and tetracycline resistance, underpinned by the *gyrA* Thr-86-Ile substitution and *tet(O),* has been reported in *Campylobacter* from broilers and retail poultry meat, including a recent slaughterhouse and retail survey in which resistance to these classes predominated [37]. Turkeys, a less studied reservoir, show a comparable pattern, with tetracycline resistance reported to be higher in *C. coli* than in C. jejuni [38], mirroring the species difference observed in our isolates. Beyond live birds, contamination and recontamination during processing steps such as defeathering and evisceration have been highlighted in broiler and turkey slaughterhouses [39], and occupational contact with pigeons and turkeys at live poultry shops may also contribute to human exposure [40]. While our current analysis was restricted to targeted resistance phenotypes and determinants, incorporating whole-genome sequencing or Multilocus Sequence Typing (MLST) in future surveillance will be essential to track high-risk lineages and transmission pathways across diverse livestock host species [25].

The *cmeABC* efflux system was widely detected across isolates, consistent with its roles as a conserved multidrug efflux system that contributes to baseline resistance levels rather than acting as a primary determinant of resistance [12,41,42]. In the present study, all three components of the cmeABC operon were consistently detected at high frequencies in *C. jejuni* (>94%), whereas *C. coli* isolates exhibited lower detection rates for *cmeB* (27.3%) and *cmeC* (63.6%). Because *cmeA*, *cmeB*, and *cmeC* function as a single operon, lower PCR detection in *C. coli* is more likely attributable to sequence variation or point mutations at the primer-binding sites rather than actual gene deletion, consistent with the higher genomic diversity and sequence heterogeneity reported in *C. coli* compared with *C. jejuni* [43]. Nevertheless, as sequencing of these operon regions was not conducted, these findings in *C. coli* should be interpreted with caution, given the limited number of isolates (n = 11).

Virulence gene profiles differed markedly between species. *C. jejuni* showed consistent carriage of adhesion (*cadF*) and motility (*flaA*, *flhA*), and the complete *cdtABC* cluster in almost all isolates. These findings were consistent with the conserved virulence potential of poultry-associated *C. jejuni* and align with whole-genome data from southern Thai isolates and regional reports from poultry systems, indicating conserved virulence potential of *C. jejuni* [8,16,17,44]. In contrast, *C. coli* appeared to have slightly heterogeneous virulence profiles, including partial or complete absence of the *cdt* operon, consistent with documented differences in virulence profiles between the two species [15]. From a One Health perspective, the high prevalence of key virulence genes, particularly in *C. jejuni*, suggests that small-scale flocks may serve as a reservoir of potentially pathogenic *Campylobacter* [15].

Farm-level factors were associated with *Campylobacter* occurrence, including larger flock size, inadequate manure management, and antimicrobial use. Larger flocks likely increase contact rates and contamination load, leading to greater transmission and persistence. These findings highlight the importance of farm management and environmental exposure in driving flock colonization, consistent with the nature of faecal-oral transmission of Campylobacter and findings of previous observations in poultry systems [28,29]. The association with AMU likely reflects underlying disease pressure and suboptimal biosecurity conditions, rather than a direct effect on Campylobacter colonization [45]. Interestingly, AMR was primarily associated with AMU in our study, particularly tetracycline. Given that quinolone and fluoroquinolone resistance was already widespread, co-resistance was largely driven by its tetracycline component, suggesting selection for *tet(O)*-mediated resistance. Other associations, such as owner age and farm location, should be interpreted cautiously, as they likely reflect underlying management practices rather than direct causal effects. The findings were consistent with studies from similar small-scale settings in the Mekong Delta of Vietnam, where frequent, heterogeneous, and often untargeted AMU has been linked to high AMR prevalence at the farm level [28,29,46]. Overall, the findings indicate that *Campylobacter* occurrence is mainly shaped by farm management and biosecurity, whereas resistance patterns are more strongly influenced by AMU. Therefore, strengthening biosecurity practices alongside antimicrobial stewardship, in line with national efforts to control AMR, is essential for reducing the burden of resistant *Campylobacter* in smallholder systems.

This study has several limitations. First, due to the observational design of the study, the observed associations between risk factors, biosecurity, AMU, and AMR cannot establish direct cause-and-effect relationships. Second, the study was conducted within a specific region in southern Thailand, which may limit the geographical generalizability of our findings to other poultry production systems or regions with different management and climatic conditions. Third, although we analyzed 358 samples across 36 farm visits, the study involved a small number of farms (n = 12) and the sample size of *C. coli* was relatively small, which may limit the statistical power of the observations. While we screened variables carefully using univariable analysis and AIC to avoid overfitting, this small farm sample size may still limit the precision of farm-level estimates and how well our findings apply to other regions. Fourth, although fluoroquinolone resistance was high, the proportion of MDR isolates remained low. This is attributable to the restricted class coverage of our panel (representing only three antimicrobial classes), combined with the complete absence of macrolide resistance, which limited the classification of isolates as MDR. Fifth, AMU was recorded qualitatively at the farm level, meaning that the intensity of use and selection pressure could not be formally quantified; thus, future studies incorporating quantitative and longitudinal AMU data would help clarify these relationships. Finally, detection of resistance determinants relied on band-based PCR detection, which may not capture the full diversity of resistance mechanisms. Therefore, taken together, these limitations highlight the value of a sequencing-based approach and improved AMU data to better understand AMR mechanisms and the interpretation of genotype-phenotype relationships.

## 4. Materials and Methods

### 4.1. Study Design and Study Area

This study employed a longitudinal observational design, lasting between March and June 2025, focusing on small-scale chicken farms in Nakhon Si Thammarat province, southern Thailand. The province is home to approximately 1.53 million people, with a chicken population of approximately 5.96 million, representing the largest human population and a substantial chicken population among the southern provinces of Thailand, making it an epidemiologically relevant setting for *Campylobacter* surveillance in poultry [47].

Small-scale chicken farms were defined in this study as production units housing fewer than 500 birds of indigenous or local breeds reared primarily for meat production (and local sale/household consumption) under low-input, non-mechanized management systems, including manual feeding and watering. These indigenous birds typically have a production cycle of around 14–23 weeks before slaughter [48,49]. This operational definition was adapted to reflect the predominant smallholder poultry production systems in southern Thailand, where flock sizes are typically household-based up to approximately 100 birds per production cycle [50,51,52]. These characteristics are consistent with regional descriptions of backyard and small-scale indigenous chicken production systems in Southeast Asia, commonly associated with limited biosecurity measures and heterogeneous AMU practices [53].

### 4.2. Sample Collection

The sample size was estimated based on a cluster sampling design, with individual farms serving as the sampling unit. A repeated cross-sectional study design at the farm level was employed in this study, with 12 farms sampled at three different time points across a single production cycle. At each farm and each time point, 10 different individual chickens were randomly selected from different areas within the housing unit to ensure representative sampling across the flock. Cloacal swab samples were collected at three pre-defined time points within the production cycle. The first visit (Time 1) was ~3 months of age, the second visit (Time 2) was ~4.5 months of age, and the third visit (Time 3) was ~6 months of age. Samples were collected using sterile cotton swabs, which were gently inserted into the cloaca and immediately transferred into Cary Blair transport medium (CITOTEST, Nanjing, China). All samples were placed in a cool box at approximately 4 °C and transported to the laboratory within 6 h of collection for microbiological processing.

### 4.3. Isolation and Identification of Campylobacter spp.

#### 4.3.1. Isolation

*Campylobacter* spp. were isolated using a selective culture approach adapted from previously described methods [26]. Upon arrival at the laboratory, swab samples were directly inoculated onto modified charcoal cefoperazone deoxycholate agar (mCCDA; OXOID, Hampshire, UK) and incubated under microaerobic conditions (5% O_2_, 10% CO_2_, and 85% N_2_), generated using a gas-generating sachet system (Mitsubishi Gas Chemicals, Tokyo, Japan), at 42 °C for 48 h. Colonies exhibiting typical *Campylobacter* morphology grey, smooth, flat, and spreading with a metallic sheen were subcultured onto Columbia blood agar (CBA; OXOID, Hampshire, UK) and incubated under identical microaerobic conditions at 42 °C for a further 24 h to obtain pure cultures. A single representative colony per sample was selected for downstream analysis.

#### 4.3.2. DNA Extraction

Genomic DNA was extracted from fresh bacterial cultures using the Presto™ Mini gDNA Bacteria Kit (Geneaid, Taipei, Taiwan) according to the manufacturer’s instructions. Prior to extraction, isolates stored at −80 °C were revived by subculture on Columbia blood agar at 42 °C for 24 h under microaerobic conditions to ensure viable and actively growing cells. A loopful of fresh colonies (approximately 10^8^–10^9^ CFU) was used as the starting material for each extraction. Extracted DNA was eluted in 100 µL preheated elution buffer and stored at −20 °C until further use. DNA purity and concentration were assessed spectrophotometrically using a NanoDrop One instrument (Thermo Fisher Scientific, Waltham, MA, USA); samples with A_260_/A_280_ ratios of approximately 1.8 and A_260_/A_230_ ratios between 2.0 and 2.2 were considered acceptable for downstream PCR analysis.

#### 4.3.3. Multiplex PCR for Identification of *Campylobacter* spp.

Species-level identification of *Campylobacter* isolates was performed using a multiplex PCR assay targeting species-specific gene markers, as described by Wang et al. [54]. Briefly, amplification of the *hipO* gene (323 bp) served as a marker for *C. jejuni*, the *glyA* gene (126 bp) for *C. coli*, and the 23S rRNA gene (650 bp) for genus-level confirmation of *Campylobacter* spp. Primer sequences and amplicon sizes are detailed in Table A1. PCR reactions were performed in a total volume of 25 µL under the following thermal cycling conditions: initial denaturation at 95 °C for 5 min; 30 cycles of denaturation at 95 °C for 30 s, annealing at 59 °C for 30 s, and extension at 72 °C for 30 s; followed by a final extension at 72 °C for 5 min. Amplification products were resolved by electrophoresis on 1.5% agarose gels in 0.5× TBE buffer and visualised under ultraviolet illumination. Reference strains *C. jejuni* ATCC 33560 and *C. coli* ATCC 33559 were included as positive controls in all PCR assays.

### 4.4. Antimicrobial Susceptibility Testing

The minimum inhibitory concentrations (MICs) of six antimicrobial agents were determined for all confirmed *Campylobacter* isolates using the agar dilution method, in accordance with the Clinical and Laboratory Standards Institute guidelines [55]. Mueller Hinton agar (OXOID, Basingstoke, UK) supplemented with 5% defibrinated sheep blood (National Laboratory Animal Center, Nakhon Pathom, Thailand) was prepared and incorporated with serial two fold dilutions of each antimicrobial agent across the following concentration ranges: ciprofloxacin (CIP, from 0.03 to 128 µg/mL), nalidixic acid (NAL, from 0.12 to 1024 µg/mL), enrofloxacin (ENR, from 0.03 to 128 µg/mL), erythromycin (ERY, from 0.25 to 1024 µg/mL), tylosin (TYL, from 0.25 to 1024 µg/mL), and tetracycline (TET, from 0.12 to 256 µg/mL). All antimicrobial compounds were obtained from Sigma-Aldrich (St. Louis, MO, USA). Resistance was defined using the specific veterinary clinical breakpoints for *Campylobacter* spp. of CLSI VET01S (2024), CIP (≥4 µg/mL), NAL (≥32 µg/mL), ERY (≥32 µg/mL), and TET (≥16 µg/mL) [55]. Because VET01S does not provide specific criteria for ENR and TYL in *Campylobacter* spp., operational cut-off values of ≥4 µg/mL (for ENR) and ≥32 µg/mL (for TYL) were derived from the corresponding CIP and ERY criteria, respectively, following the approach of a previous study in Thailand. All criteria were applied identically to both *C. jejuni* and *C. coli* [26]. Isolates exhibiting resistance to at least one antimicrobial agent in three or more antimicrobial classes were classified as multidrug resistant (MDR) [56].

For inoculum preparation, a loopful of each 24-h culture was suspended in sterile normal saline and the turbidity adjusted to a 0.5 McFarland standard, equivalent to approximately 1.0 × 10^8^ CFU/mL. The standardised suspension was then applied to the surface of the prepared agar plates using a 48-pin replicator device. To verify the absence of contamination and to confirm the absence of antimicrobial carryover between plates, two drug-free control plates were included at the beginning and end of each inoculation run. Inoculated plates were incubated at 42 °C for 48 h under microaerobic conditions (5% O_2_, 10% CO_2_, and 85% N_2_). The MIC was defined as the lowest concentration of each antimicrobial agent at which no visible bacterial growth was observed. The reference strain *C. jejuni* ATCC 33560 was included in every batch as a quality control, with acceptable MIC ranges validated against CLSI performance standards [55].

### 4.5. Detection of AMR Genes

Key genetic determinants associated with AMR in *Campylobacter* spp. were investigated by PCR, targeting genes and point mutations linked to resistance to the three principal antimicrobial classes examined in this study. For quinolone resistance, the Thr-86-Ile substitution in QRDR of the *gyrA* gene was detected using species-specific primer sets for *C. jejuni* and *C. coli*, as the sequences of this gene differ sufficiently between the two species to require separate assays [32,33]. For macrolide resistance, point mutations at positions A2075G and A2074C in domain V of the 23S rRNA gene were detected using a mismatch amplification mutation assay (MAMA-PCR), as described by Alonso et al. (2005) [36]. For tetracycline resistance, the presence of the plasmid-encoded *tet(O)* gene was assessed using primers reported by Gibreel et al. (2004) [35]. In addition, the three component genes of the *CmeABC* multidrug efflux pump (*cmeA*, *cmeB,* and *cmeC*) were examined using species-specific primer sets for *C. jejuni* and *C. coli*, given the sequence divergence of these genes between species [41,57]. Primer sequences, amplicon sizes, and annealing temperatures for all targets are detailed in Table A2.

All PCR reactions were performed in a total volume of 25 µL containing 1× PCR buffer, 0.2 mM dNTPs, 1.5 mM MgCl_2_, 10 µM of each primer, 5 U of Taq DNA polymerase, and approximately 20 ng of template DNA. Thermal cycling conditions were as follows: initial denaturation at 94 °C for 5 min; 30 cycles of denaturation at 94 °C for 1 min, primer-specific annealing at the temperatures listed in Table A2 for 1 min, and extension at 72 °C for 1 min; followed by a final extension at 72 °C for 5 min. Amplification products were resolved by electrophoresis on 1.5% agarose gels in 0.5× TBE buffer at 80 V, stained with SYBR Safe (Invitrogen, Carlsbad, CA, USA), and visualised under ultraviolet illumination. Amplicon sizes were confirmed by comparison with a 100 bp DNA ladder. Reference strains *C. jejuni* ATCC 33560 and *C. coli* ATCC 33559 were included as positive controls in all PCR runs, and nuclease-free water was used as a negative control.

### 4.6. Detection of Virulence Genes by PCR

The presence of six virulence-associated genes (*flaA*, *flhA*, *cadF*, *cdtA*, *cdtB*, *cdtC*) was investigated in all confirmed *Campylobacter* isolates by PCR. Species-specific primer sets were used as follows: *cadF*, *cdtA*, *cdtB, cdtC*, *flaA*, and *flhA* [58,59,60,61]. Primer sequences, amplicon sizes, and annealing temperatures are summarised in Table A3. Concurrent detection of *cdtA*, *cdtB*, and *cdtC* in the same isolate was defined as the presence of the complete *cdt* gene cluster. PCR amplification was performed under the reaction conditions described in Section 4.5, with primer-specific annealing temperatures listed in Table A3. Amplification products were resolved on 1.5% agarose gels in 0.5× TBE buffer at 80 V, stained with SYBR Safe (Invitrogen, Carlsbad, CA, USA), and visualised under ultraviolet illumination. Amplicon sizes were confirmed against a 100 bp DNA ladder. Reference strains *C. jejuni* ATCC 33560 and *C. coli* ATCC 33559 were included as positive controls, and nuclease-free water served as a negative control in all PCR assays.

### 4.7. Data Collection on AMU and Farm-Level Risk Factors

Farm-level data were collected at each sampling visit using a structured questionnaire administered through face-to-face interviews with farm owners/managers. The questionnaire was adapted from a previously validated survey instrument used in poultry farms in the same study context and region, and is provided in the Supplementary File S1 [62]. Two broad categories of information were collected as follows: (i) general farm and flock characteristics, and (ii) AMU patterns at each time point. General farm and flock characteristics were recorded at the first visit and included farm location; years of experience in chicken farming; flock size and breed type; housing area (m^2^); floor type (concrete or soil/earth); litter or bedding material; drinking water source and system; presence of other animal species on the farm; and cleaning and disinfection practices applied between production cycles. AMU was defined as the administration of any product containing identifiable antimicrobial active ingredients, regardless of formulation, route, or intended purpose. Products with unverifiable antimicrobial content were excluded. AMU data were collected at each sampling visit to ensure temporal alignment with microbiological sampling. To minimise recall bias, farm owners were asked to retain antimicrobial product packaging, and photographic records were obtained for cross-verification. AMU was assessed qualitatively as binary or categorical variables, with the information recorded including antimicrobial class, active ingredient, route of administration, purpose of use, treatment duration, and reported dosage.

### 4.8. Data Analyses

All statistical analyses were performed using R software (version 4.5.3, R Core Team, 2026). Descriptive statistics were applied to summarise farm characteristics, AMU patterns, and the prevalence of *Campylobacter* spp., AMR phenotypes, resistance-associated genes, and virulence-associated genes. Categorical variables were presented as frequencies and percentages.

To account for unequal probabilities resulting from variations in flock sizes across farms and sampling visits, stratum-specific sampling weights (W_ij_) were applied to obtain representative population-level prevalence and resistance estimates. The sampling weight (W_ij_) for a bird from farm i at visit j was calculated as the inverse of its selection probability (p_ij_), with the formula W_ij_ = 1/p_ij_ = N_ij_/n_ij_, where N_ij_ is the flock size of farm i at visit j, and n_ij_. For example, if 10 birds were sampled from a flock of 120 birds at farm A during Visit 1, the selection probability (p_ij_) for a bird in that flock was 10/120 = 0.083 (8.3%). Its corresponding sampling weight was W_A,1_ = 120/10 = 12, indicating that each sampled bird represented 12 birds in the target population. It means a selected bird was considered to represent 12 birds on farm A. Without weighting, birds from smaller flocks would be overrepresented in the overall estimates. Weighted prevalence estimates and standard errors were computed using the *survey* package in R [63].

Agreement between phenotypic and genotypic resistance: Agreement between phenotypic resistance (MIC-based classification) and the presence of corresponding resistance-associated genes or mutations was assessed using Cohen’s kappa statistic [64]. Agreement was evaluated for each phenotype–genotype pair: ciprofloxacin, nalidixic acid, and enrofloxacin resistance versus the presence of the *gyrA* Thr-86-Ile mutation; while erythromycin and tylosin resistance versus the 23S rRNA mutations A2075G and A2074C; and tetracycline resistance versus *tet(O)*. Kappa values were interpreted as: slight (κ < 0.20); fair (κ: 0.21–0.40); moderate (κ: 0.41–0.60); substantial (κ: 0.61–0.80); almost perfect (κ > 0.80) [65]. Statistical significance was assessed using Fisher’s exact test or chi-square test as appropriate. Calculations were performed using the *epiR* package in R [66].

Risk factors for the occurrence and AMR of *Campylobacter* spp.: Two separate mixed-effects logistic regression models were developed with different outcome variables. The first model examined farm-level factors associated with the occurrence of *Campylobacter* spp., using cloacal sample positivity as the binary outcome. The second model assessed factors associated with AMR among *Campylobacter* isolates. Based on the observed laboratory resistance patterns, co-resistance to ciprofloxacin and tetracycline (CIP–TET), the predominant phenotype, was defined as the binary outcome variable for this model. In both models, the farm-visit event (36 distinct clusters: 12 farms × 3 sampling visits) was included as a random effect to account for potential clustering within both individual farms and sampling timepoints. A total of twenty explanatory variables were considered, including sampling time, farm location, owner demographics (age, education, experience), flock and housing characteristics (flock size, pen area, litter type, heating, floor type), presence of other animals (cattle, dogs), biosecurity practices (manure management, hand washing, feeder cleaning), water source, and farm-level AMU (active antimicrobial agents). First, univariable mixed-effects logistic regression was performed to screen candidate variables (*p* < 0.20). Multivariable models were then constructed using a stepwise selection procedure based on Akaike’s Information Criterion (AIC). Model diagnostics and goodness-of-fit were assessed by comparing AIC values, and the model yielding the lowest AIC value was retained as the final multivariable model. Variables with a *p*-value < 0.05 in multivariable models were considered significantly associated with either *Campylobacter* occurrence or antimicrobial resistance.

## 5. Conclusions

This longitudinal study integrated phenotypic resistance, resistance and virulence gene profiling, and farm-level factors to characterize *Campylobacter* spp. in small-scale chicken farms in southern Thailand. *C. jejuni* predominated over *C. coli*, and weighted prevalence increased across the production cycle within the sampled flocks. High quinolone and fluoroquinolone resistance in *C. jejuni* showed substantial agreement with the *gyrA* Thr-86-Ile mutation, and tetracycline resistance showed moderate agreement with *tet(O)*; together with the frequent carriage of the selected virulence genes, these findings indicate a substantial resistance burden and intact colonization potential in this specific study population. In contrast, erythromycin susceptibility was fully preserved despite near-universal A2075G detection, an unexpected genotype–phenotype discordance that should be interpreted with caution and confirmed by sequencing in future surveillance.

Farm-level factors associated with *Campylobacter* occurrence differed from those associated with ciprofloxacin–tetracycline co-resistance, suggesting that pathogen control and resistance control may require complementary interventions: improved manure management and biosecurity to reduce bacterial circulation, and antimicrobial stewardship to limit resistance selection. Given the observational design and limited farm sample size, these observed associations are descriptive and exploratory, and do not imply causal relationships or apply directly beyond the sampled farms and geographic area. Overall, the results support a One Health approach combining targeted stewardship with feasible biosecurity measures; further studies with quantitative antimicrobial-use data, whole-genome sequencing, and cross-species sampling are needed to clarify transmission and resistance dissemination across animal, human, and environmental interfaces.

## Figures and Tables

**Table 1:** MIC distributions of *C. jejuni* and *C. coli* isolates, with color-coded susceptibility categories and breakpoint indicators.

Antimicrobial Agents	0.03	0.06	0.12	0.25	0.5	1	2	4	8	16	32	64	128	256	512	1024	S (%)	I (%)	R (%)
CIP (S ≤ 1, I = 2, R ≥ 4 µg/mL)																			
*C. jejuni* (142)	0	0	5	8	9	9	9	16	36	112	141	142	142				9 (6.34)	0 (0.00)	133 (93.66)
*C. coli* (11)	0	0	0	1	1	2	2	3	3	3	7	11	11				2 (18.18)	0 (0.00)	9 (81.82)
NAL (S ≤ 16, R ≥ 32 µg/mL)																			
*C. jejuni* (142)			0	0	0	1	5	10	10	11	14	41	118	138	142	142	11 (7.75)	0 (0.00)	131 (92.25)
*C. coli* (11)			0	0	0	0	0	2	3	3	3	3	10	11	11	11	3 (27.27)	0 (0.00)	8 (72.73)
ENR (S ≤ 1, I = 2, R ≥ 4 µg/mL)																			
*C. jejuni* (142)	0	4	6	9	9	9	37	136	142	142	142	142	142				9 (6.34)	28 (19.72)	105 (73.94)
*C. coli* (11)	0	2	2	3	3	3	3	5	10	10	10	10	10				3 (27.27)	0 (0.00)	8 (72.73)
ERY (S ≤ 8, I = 16, R ≥ 32 µg/mL)																			
*C. jejuni* (142)				0	51	122	141	142	142	142	142	142	142	142	142	142	142 (100)	0 (0.00)	0 (0.00)
*C. coli* (11)				0	2	4	8	11	11	11	11	11	11	11	11	11	11 (100)	0 (0.00)	0 (0.00)
TYL (S ≤ 8, I = 16, R ≥ 32 µg/mL)																			
*C. jejuni* (142)				0	0	0	1	42	111	141	142	142	142	142	142	142	111 (78.17)	30 (21.13)	1 (0.7)
*C. coli* (11)				0	0	1	1	3	4	8	11	11	11	11	11	11	4 (36.36)	4 (36.36)	3 (27.27)
TET (S ≤ 4, I = 8, R ≥ 16 µg/mL)																			
*C. jejuni* (142)			15	20	25	28	29	36	53	76	107	135	142	142	142		36 (25.35)	17 (11.97)	89 (62.68)
*C. coli* (11)			0	1	2	5	5	5	6	7	8	11	11	11	11		5 (45.45)	1 (9.09)	5 (45.45)

MIC distributions are presented as cumulative percentages. Susceptibility classifications were determined based on the CLSI VET01S (2024) guidelines. Color legend: dark blue: susceptible (S); light blue: intermediate (I); grey: resistant (R). Abbreviations: CIP: ciprofloxacin; NAL: nalidixic acid; ENR: enrofloxacin; ERY: erythromycin; TYL: tylosin; TET: tetracycline. Resistance estimates are presented as weighted prevalence for *C. jejuni* and crude prevalence for *C. coli*.

**Table 2:** Distribution of AMR profiles among *Campylobacter* isolates.

	No. of Antimicrobial Classes	AMR Profile	Resistant Isolates, % (n)
*C. jejuni* (n = 142)	3 (MDR)	CIP ENR NAL TYL TET	0.70 (1)
	2	CIP ENR NAL TET	45.07 (64)
	2	CIP NAL TET	13.38 (19)
	2	ENR NAL TET	0.70 (1)
	2	CIP TET	1.41 (2)
	2	CIP ENR NAL	26.76 (38)
	1	CIP NAL	4.93 (7)
	1	CIP	1.41 (2)
	1	TET	0.70 (1)
	0	Susceptible to all tested antimicrobials	4.93 (7)
	**Total**		**100 (142)**
*C. coli* (n = 11)	3 (MDR)	CIP ENR NAL TYL TET	9.09 (1)
	2	CIP ENR NAL TET	27.27 (3)
	2	CIP ENR NAL TYL	18.18 (2)
	2	CIP ENR NAL	18.18 (2)
	1	CIP	9.09 (1)
	1	TET	9.09 (1)
	0	Susceptible to all tested antimicrobials	9.09 (1)
	**Total**		**100 (11)**

Abbreviations: MDR: multidrug resistance; CIP: ciprofloxacin; NAL: nalidixic acid; ENR: enrofloxacin; TYL: tylosin; TET: tetracycline.

**Table 3:** Distribution of AMR genes in *Campylobacter* isolates.

Genes	*C. jejuni* (n = 142)			*C. coli* (n = 11)		
	Time 1 (n = 44)	Time 2 (n = 48)	Time 3 (n = 50)	Time 1 (n = 1)	Time 2 (n = 3)	Time 3 (n = 7)
*gyrA*	92.70 ± 0.04	94.32 ± 0.04	89.18 ± 0.05	100.00	100.00	85.71 ± 0.14
A2075G	100.00	100.00	100.00	100.00	66.67 ± 0.33	100.00
A2074C	3.04 ± 0.03	5.68 ± 0.04	0.00	0.00	0.00	57.14 ± 0.20
*cmeA*	100.00	100.00	100.00	100.00	100.00	100.00
*cmeB*	100.00	100.00	100.00	100.00	0.00	28.57 ± 0.18
*cmeC*	100.00	100.00	100.00	0.00	33.33 ± 0.33	85.71 ± 0.14
*tet(O)*	78.12 ± 5.57	79.55 ± 0.06	83.23 ± 0.04	0.00	33.33 ± 0.33	42.86 ± 0.20

Abbreviations: SE: standard error; n: number of isolates; *gyrA*: DNA gyrase gene; A2075G/A2074C: 23S rRNA mutations; *cmeA*, *cmeB*, *cmeC*: efflux pump genes; *tet(O)*: tetracycline resistance gene. Prevalence estimation: weighted estimates (mean ± SE) are reported for *C. jejuni*; crude prevalence is reported for *C. coli* due to small sample size (n = 11).

**Table 4:** Agreement between phenotypic and genotypic AMR in *Campylobacter* isolates (n = 153 isolates).

Active Antimicrobial Agents	N_P_	Gene	N_G_	P+/G+	P+/G−	P−/G+	P−/G−	Kappa (*κ*)	*p*-Value	Agreement
Quinolones and fluoroquinolones										
Ciprofloxacin	142	*gyrA* mutation	143	140	2	3	8	0.744	<0.001 ***	Substantial
		*cmeA*	153	142	0	11	0	NC	NC	NC
		*cmeB*	145	135	7	10	1	0.048	0.458	Slight
		*cmeC*	149	140	2	9	2	0.237	0.026 *	Fair
Nalidixic acid	139	*gyrA* mutation	143	137	2	6	8	0.639	<0.001 ***	Substantial
		*cmeA*	153	139	0	14	0	NC	NC	NC
		*cmeB*	145	133	6	12	2	0.123	0.158	Slight
		*cmeC*	149	138	1	11	3	0.305	0.002 **	Fair
Enrofloxacin	113	*gyrA* mutation	143	111	2	32	8	0.241	<0.001 ***	Fair
		*cmeA*	153	113	0	40	0	NC	NC	NC
		*cmeB*	145	107	6	38	2	−0.004	1	Poor
		*cmeC*	149	112	1	37	3	0.093	0.055	Slight
Macrolides										
Erythromycin	0	A2075G	152	0	0	152	1	NC	NC	NC
		A2074C	7	0	0	7	146	NC	NC	NC
		*cmeA*	153	0	0	153	0	NC	NC	NC
		*cmeB*	145	0	0	145	8	NC	NC	NC
		*cmeC*	149	0	0	149	4	NC	NC	NC
Tylosin	4	A2075G	152	4	0	148	1	0.000	0.869	Slight
		A2074C	7	4	0	3	146	0.718	<0.001 ***	Substantial
		*cmeA*	153	4	0	149	0	NC	NC	NC
		*cmeB*	145	1	3	144	5	−0.039	<0.001 ***	Poor
		*cmeC*	149	4	0	145	4	0.001	0.740	
Tetracyclines										
Tetracycline	94	*tet(O)*	112	86	8	26	33	0.503	<0.001 ***	Moderate
		*cmeA*	153	94	0	59	0	NC	NC	NC
		*cmeB*	145	90	4	55	4	0.030	0.495	Slight
		*cmeC*	149	92	2	57	2	0.015	0.634	Slight

Abbreviations: N_P_, number tested by MIC; N_G_, number tested for gene detection; P+/G+, phenotype-positive/genotype-positive; P+/G−, phenotype-positive/genotype-negative; P−/G+, phenotype-negative/genotype-positive; P−/G−, phenotype-negative/genotype-negative. Significance: * *p* < 0.05; ** *p* < 0.01; *** *p* < 0.001. NC (not computable): due to zero cell counts (e.g., universal gene presence or absence of phenotypic resistance), preventing kappa estimation.

**Table 5:** Univariable and multivariable logistic regression analyses identifying factors associated with *Campylobacter* positivity in small-scale chicken farms in southern Thailand (n = 358 samples, 151 positive events).

Factors	Positive/Total (%)	Univariable OR	95% CI	*p*-Value	Adjusted OR (aOR)	95% CI	*p*-Value
Location (baseline = Hua Taphan)	61/178 (34.3)						
Pho Thong	90/180 (50.0)	1.92	1.26–2.94	0.003			
Owner age (baseline = ≤50)	65/208 (31.2)						
>50 years	86/150 (57.3)	2.96	1.92–4.59	<0.001			
Education (baseline = post-secondary)	36/120 (30.0)						
Secondary	115/238 (48.3)	2.18	1.38–3.50	0.001			
Flock size (baseline = ≤60)	53/208 (25.5)						
> 60 birds	98/150 (65.3)	5.51	3.51–8.78	<0.001	3.15	1.74–5.85	<0.001
Pen area (baseline = ≤300)	67/208 (32.2)						
>300 m^2^	84/150 (56.0)	2.68	1.74–4.15	<0.001			
Litter type (baseline = none)	109/300 (36.3)						
Wood	16/28 (57.1)	2.34	1.07–5.23	0.034			
Rice husk	26/30 (86.7)	11.39	4.30–39.36	<0.001			
Heating system (baseline = No)	125/328 (38.1)						
Present	26/30 (86.7)	10.56	4.00–36.40	<0.001			
Cattle present (baseline = No)	72/210 (34.3)						
Yes	79/148 (53.4)	2.19	1.43–3.39	<0.001			
Manure management (baseline = No)	11/120 (9.1)						
Inadequate	140/238 (58.8)	14.16	7.52–29.17	<0.001	9.65	4.84–20.92	<0.001
Feeder cleaned (baseline = No)	22/90 (24.4)						
Yes	129/268 (48.1)	2.87	1.70–5.00	<0.001			
Antimicrobial use (baseline = No)	108/308 (35.1)						
Yes	43/50 (86.0)	11.38	5.25–28.45	<0.001	3.85	1.36–12.13	0.015

Abbreviations: OR, odds ratio; CI, confidence interval.

**Table 6:** Univariable and multivariable logistic regression analyses identifying factors associated with AMR in *Campylobacter* isolates from small-scale chicken farms in southern Thailand (n = 151 positive samples, 91 CIP-TET co-resistant events).

Factors	CIP–TET Co-Resistant/Total (%)	Univariable OR	95% CI	*p*-Value	Adjusted OR (aOR)	95% CI	*p*-Value
Sampling time (baseline = Time 2)	26/51 (51.0)						
Time 1	34/45 (75.6)	2.97	1.26–7.33	0.015			
Time 3	31/55 (56.4)	1.24	0.58–2.68	0.579			
Location (baseline = Pho Thong)	49/90 (54.4)						
Hua Taphan	42/61 (68.9)	1.85	0.94–3.71	0.077	9.00	2.89–32.42	<0.001
Owner age (years) (baseline = >50 years)	40/86 (46.5)						
≤50 years	51/65 (78.5)	4.19	2.06–8.90	<0.001	13.34	4.88–44.05	<0.001
Education level (baseline = secondary)	60/115 (52.2)						
post-secondary	31/36 (86.1)	5.68	2.23–17.57	<0.001			
Farming experience (years) (6–10 years)	48/82 (58.5)						
>10 years	16/37 (43.2)	0.54	0.24–1.24	0.124			
<5years	27/32 (84.4)	3.83	1.43–12.18	0.012			
Litter type (baseline = None)	58/109 (53.2)						
Wood	9/16 (56.2)	1.13	0.39–3.37	0.820			
Rice husk	24/26 (92.3)	10.55	2.94–67.69	0.002			
Heating in pen (baseline = No)	67/125 (53.6)						
Yes	24/26 (92.3)	10.39	2.91–66.39	0.002			
Other animals on farm (baseline = No)	39/49 (79.6)						
Yes	52/102 (51.0)	0.27	0.12–0.57	0.001			
Cattle presence (baseline = No)	57/72 (79.2)						
Yes	34/79 (43.0)	0.20	0.09–0.40	<0.001			
Concrete floor (baseline = No)	47/94 (50.0)						
Yes	44/57 (77.2)	3.38	1.65–7.29	0.001			
Feeder cleaned (baseline = No)	9/22 (40.9)						
Yes	82/129 (63.6)	2.52	1.01–6.53	0.050			
Tetracycline use (baseline = No)	57/97 (58.8)						
Yes	34/54 (63.0)	1.19	0.60–2.39	0.613	5.67	1.97–18.46	0.002

Abbreviations: OR, odds ratio; CI, confidence interval.

## Data Availability

The original contributions presented in this study are included in the article/Supplementary Material. Further inquiries can be directed to the corresponding author.

## Supplementary Materials

The following supporting information can be downloaded at https://www.mdpi.com/article/10.3390/antibiotics15080756/s1: File S1: questionaires used in this study.

## Abbreviations

The following abbreviations are used in this manuscript:

aOR	Adjusted odds ratio
AMR	Antimicrobial resistance
AMU	Antimicrobial usage
ATCC	American Type Culture Collection
bp	Base pairs
CFU	Colony-forming unit
CI	Confidence interval
CIP	Ciprofloxacin
CLSI	Clinical and Laboratory Standards Institute
DNA	Deoxyribonucleic acid
dNTP	Deoxynucleotide triphosphate
ENR	Enrofloxacin
ERY	Erythromycin
FAO	Food and Agriculture Organization of the United Nations
MAMA-PCR	Mismatch amplification mutation assay PCR
mCCDA	Modified charcoal cefoperazone deoxycholate agar
MDR	Multidrug resistance
MIC	Minimum inhibitory concentration
MLST	Multilocus sequence typing
NAL	Nalidixic acid
NG	Number of isolates tested for gene detection
NP	Number of isolates tested by MIC
OR	Odds ratio
PCR	Polymerase chain reaction
QRDR	Quinolone resistance-determining region
R	Resistant
rRNA	Ribosomal ribonucleic acid
S	Susceptible
SE	Standard error
TBE	Tris-borate-EDTA
TET	Tetracycline
TYL	Tylosin
WU ACUC	Walailak University Institutional Animal Care and Use Committee
WU IBC	Walailak University Institutional Biosafety Committee

## Appendix A

**Table A1:** Primers used for genus confirmation and species identification of *Campylobacter* isolates.

Gene Target	Primers	Primer Sequence (5′–3′)	Amplicon Size (bp)
23S rRNA	23S-F	TATACCGGTAAGGAGTGCTGGAG	650
	23S-R	ATCAATTAACCTTCGAGCACCG	
*C*. *jejuni hipO*	CJ-F	ACTTCTTTATTGCTTGCTGC	323
	CJ-R	GCCACAACAAGTAAAGAAGC	
*C*. *coli glyA*	CC-F	GTAAAACCAAAGCTTATCGTG	126
	CC-R	TCCAGCAATGTGTGCAATG	

**Table A2:** Primers used for detection of antimicrobial resistance-associated genes in *Campylobacter* isolates.

Gene Target	Primers	Primer Sequence (5′–3′)	Amplicon Size (bp)	Annealing Temperature (°C)	Reference
*C. jejuni gyrA* and *gyrA* mutation (Thr-86-Ile)	CampyMAMAgyrA1-F	TTTTTAGCAAAGATTCTGAT			[32]
	GZgyrA4-R	CAGTATAACGCATCGCAGCG	368	50	
	CampyMAMAgyrA5-R	CAAAGCATCATAAACTGCAA	265	50	
*C. coli gyrA* and *gyrA* mutation (Thr-86-Ile)	GZgyrAC*Coli*3-F	TATGAGCGTTATTATCGGTC			[33]
	GZgyrAC*Coli*4-R	GTCCATCTACAAGCTCGT TA	505	50	
	CampyMAMAgyrA8-R	TTAGGCATCGTAAACAGCCA	192	50	
23S rRNA mutation A2075G A2074C	23SRNA-F	TTAGCTAATGTTGCCCGTACCG			[36]
	ERY2075-R	TAGTAAAGGTCCACGGGGTCGC	485	59	
	ERY2074-R	AGTAAAGGTCCACGGGGTCTGG	485	59	
*tet(O)*	DMT-F	GGCGTTTTGTTTATGTGCG	559	50	[35]
	DMT-R	ATGGACAACCCGACAGAAGC			
*C. jejuni cmeA*	cmeA-F	TAGCGGCGTAATAGTAAATAAAC	435	49.8	[41]
	cmeA-R	ATAAAGAAATCTGCGTAAATAGGA			
*C. jejuni cmeB*	cmeB-F	AGGCGGTTTTGAAATGTATGTT	444	50.8	
	cmeB-R	TGTGCCGCTGGGAAAAG			
*C. jejuni cmeC*	cmeC-F	CAAGTTGGCGCTGTAGGTGAA	431	52.3	
	cmeC-R	CCCCAATGAAAAATAGGCAGAGTA			
*C. coli cmeA*	cmeA-F	TTGATGGCTAAGGCAACTTTC	771	53	[42]
	cmeA-R	CTCCAATTTCTTTAACTTCGC			
*C. coli cmeB*	cmeB-F	GACGTAATGAAGGAGAGCCA	1070	52	
	cmeB-R	CTGATCCACTCCAAGCTATG			
*C. coli cmeC*	cmeC-F	GCTTGGAGCCTTATCTTGGGA	571	57	
	cmeC-R	TGGCTCTTGCTTGAGCAAGTT			

Abbreviations: bp, base pairs; *gyrA*, DNA gyrase subunit A gene; 23S rRNA, ribosomal RNA gene; *tet(O)*, tetracycline resistance gene; *cmeA*, *cmeB*, *cmeC*, efflux pump genes.

**Table A3:** Primers used for detection of virulence-associated genes in *Campylobacter* isolates.

Gene	Primers	Sequence (5′–3′)	Product Size (bp)	Annealing Temperature (°C)	Reference
*cadF*	cadF-F	TTGAAGGTAATTTAGATAT	400	45	[58]
	cadF-R	CTAATACCTAAAGTTGAAAC			
*cdtA*	cdtA-F	CCTTGTGATGCAAGCAATC	370	55	[59]
	cdtA-R	ACACTCCATTTGCTTTCTG			
*cdtB*	cdtB-F	CAGAAAGCAAATGGAGTGTT	620	55	[60]
	cdtB-R	AGCTAAAAGCGGTGGAGTAT			
*cdtC*	cdtC-F	CGATGAGTTAAAACAAAAAGATA	182	55	[60]
	cdtC-R	TTGGCATTATAGAAAATACAGTT			
*flaA*	flaA-F	AATAAAAATGCTGATAAAACAGGTG	855	53	[60]
	flaA-R	TACCGAACCAATGTCTGCTCTGATT			
*flhA*	flhA-F	GGAAGCGGCACTTGGTTTGC	735	54	[61]
	flhA-R	GCTGTGAGTGAGATTATAGCAGC			

Abbreviations: bp, base pairs; *flaA*, flagellin gene; *flhA*, flagellar biosynthesis gene; *cadF*, fibronectin-binding protein gene; *cdtA*, *cdtB*, *cdtC*, cytolethal distending toxin genes.
