# Supplementary material for: Molecular Characteristics and Factors Associated with Antimicrobial Resistance in Campylobacter Isolates from Small-Scale Chicken Farms in Southern Thailand
Source: Antibiotics (Basel). 2026 Aug 5;15(8):756. doi: 10.3390/antibiotics15080756 (PMC13509914; doi:10.3390/antibiotics15080756)
Supplement: Supplementary file 1 [file antibiotics-15-00756-s001.zip › First Visit_EN_1.pdf]

AMR and AMU project

Questionnaire: START-OF-CYCLE			
Study Identifier	Study Site<br>Nakhon Si Thammarat	Farm ID<br>[__|__]	Flock ID<br>[__|__]

Visit date (dd/mm/yy): ____/____/____

Interviewer's name: [______________________________] Role in the project: [_____________________]

This form is to be completed within 1 day after arrival of new chickens to restock your designated target flock (day-old chicks) into the farms. If more chicks are to be purchased on subsequent days, this information will need to be provided in the mid-production questionnaire.

A. GENERAL FARM INFORMATION: (to be completed on the first visit only)

A1. Location of farming:

Village [_____________________________]

Commune [_____________________________]

District [_____________________________]

A2. GPS coordinates: Longitude [_____________________], Lattitude [______________________]

B. TARGET FLOCK

B1. No. chicks purchased [_______], No. died (in transit/on farm after arrival) [_______], No. chicks on farm today [_______].

B2. Breed of chicks (if >1 breed, indicate names of all breeds): [____________________________________]

B3. Total surfaces of areas used to keep the chicks (this refers to the area reserved to chicks during brooding period): [_______]m^2

B4. What type of litter/bedding is used for chicks:

○ Nothing ○ Rice husks ○ Straws ○ Wood shaving ○ Other, specify [________________]

B5. Do you provide heating to your chicks

○ No ○Yes

Questionnaire: START-OF-CYCLE			
**Study Identifier**	**Study Site** Nakhon Si Thammarat	**Farm ID** [__|__]	**Flock ID** [__|__]

C. OTHER ANIMALS IN THE FARM

C1. Do you now keep in your farm other **chickens in addition to the target flock**?

○ No ○ Yes; How many? [__________] chicken

C2. Indicate the **age of these other chickens** (if mixed or several flocks, indicate range):

[____|-|____] ○ Days ○ Weeks ○ Months

Purpose (click all that apply): ○ Meat ○ Eggs ○ Fighting ○ Other; Specify: [___________________]

C3. Any other animal species on the farm in addition to chickens?

○ No ○ Yes (fill below numbers of each)

Quails	[______]	Ducks	[______]
Pidgeons	[______]	Muscovy ducks	[______]
Geese	[______]	Turkeys	[______]
Pigs	[______]	Cattles/buffaloes	[______]
Dogs	[______]	Cats	[______]

Other animal species (a), Specify animal species [______________] No: [______]

Other animal species (b), Specify [______________] No: [______]

C4. Is there a fish pond/s in your farm?

○ No ○ Yes (currently not used for fish production) ○ Yes (currently used for fish production)

D. CHICKEN HOUSE/PEN STYLE DESCRIPTION AND DRINKING WATER

D1. Describe the house/pen you use to keep your chicks

○ On stilts on solid groud ○ On stilts over pond/canal/river ○ On solid ground

D2. Chicken pen/house floors are made of:

○ Concrete ○ Soil/earth ○ Metal ○ Wood ○ Metal mesh ○ Other (specify): [________________]

D3. Drinking systems available to the chicks (select all that apply):

○ Trough ○ Bowl ○ Nipple drinkers ○ Other (specify): [________________]

D4. Source of drinking water for the target flock (tick all that apply):

○ Municipal supply ○ Borehole/well ○ Rain water. ○ Pond ○ River/stream/canal

○ Other; specify [_________________]

D5. If source of drinking water for chickens other than municipal supply, do you treat it?

Questionnaire: START-OF-CYCLE			
**Study Identifier**	**Study Site**<br>**Nakhon Si Thammarat**	**Farm ID**<br>**[__|__]**	**Flock ID**<br>**[__|__]**

○ No ○ Yes, by adding chemicals ○ Yes, by boiling ○ Other; specify [________________]

E. DESCRIPTION OF THE CLEANING, WASHING, AND DISINFECTION AFTER DEPOPULATION OF PREVIOUS FLOCK

E1. How long has the pen/house where you keep your chicks been empty (i.e. time since you sold the last chickens in the house/pen used where you place your new chicks) [___________]☐ days ☐ weeks ☐ months

E2. Describe the **steps** you followed **for washing, cleaning and disinfecting** in the house/pen where you keep your chicks after moving the last flock out? (i.e. in the empty pen/house) (Tick all that apply)

○ Mucking out only (physical removal of litter/manure):

○ Washing with water only

○ Washing with water containing disinfectant

○ None of the steps above

○ Other; specify [________________]

E3. In addition to the pen/house, did you **clean**, **washed and/or disinfected feeders and drinkers**? (Tick all that apply)

○ No, nothing of the above is done.

○ The remaining feed and water is mechanically removed

○ Feeders and drinkers are washed with water

○ Feeders and drinkers are washed with water containing disinfectant

○ Other; specify [________________]

PICTURES OF TARGET FLOCK

[Farm ID] - [Flock ID] - [Start-of-cycle] - [Picture 01]

[Farm ID] - [Flock ID] - [Start-of-cycle] - [Picture 02]

[Farm ID] - [Flock ID] - [Start-of-cycle] - [Picture 03]

(*Examples: AB-01-S-01, AB-01-S-02, AB-01-S-03)*

Questionnaire: START-OF-CYCLE			
**Study Identifier**	**Study Site**<br>**Nakhon Si Thammarat**	**Farm ID**<br>**[__|__]**	**Flock ID**<br>**[__|__]**

SAMPLING RECORD

Date of sample collection: [__|__]/[__|__]/[__|__] (dd/mm/yy)

Farm Visit Time:

- ☐ **Start-of-cycle** (1 DOC)
- ☐ **Middle-of-cycle** (around 4-6 weeks)
- ☐ **End-of-cycle** (around 10 weeks)

Loại mẫu:

- ☐ **Cloacal swabs (10 chickens)**

[Farm ID] – [Flock ID] – [Sample ID]

(Example: from *AB-01-01* to *AB-01-10*)

Signature of sample collector

Full name
