# Supplementary material for: Molecular Characteristics and Factors Associated with Antimicrobial Resistance in Campylobacter Isolates from Small-Scale Chicken Farms in Southern Thailand
Source: Antibiotics (Basel). 2026 Aug 5;15(8):756. doi: 10.3390/antibiotics15080756 (PMC13509914; doi:10.3390/antibiotics15080756)
Supplement: Supplementary file 1 [file antibiotics-15-00756-s001.zip › Second Visit_EN_1.pdf]

AMR and AMU project

Questionnaire: MIDDLE-OF-CYCLE			
**Study Identifier**	**Study Site**<br>**Nakhon Si Thammarat**	**Farm ID**<br>**[__|__]**	**Flock ID**<br>**[__|__]**

Visit date (dd/mm/yy): ____/____/____

Interviewer's name: [______________________________] Role in the project: [_____________________]

This form is to be completed around 4-6 weeks of your designated target flock. You will be asked about information on the flock from the first day after arrival until 4th-6th week. All answered information/data will be kept privately, only the project members can access and analyze.

A. GENERAL FARM INFORMATION: (applied in case a different interviewee)

A1. Location of farming:

Village [______________________________]

Commune [______________________________]

District [______________________________]

A2. GPS coordinates: Longitude [_____________________], Lattitude [______________________]

B. TARGET FLOCK

B1. Number of chickens at farms changing by week.

Week	No. chickens added to the flock	Reasons for adding more chickens	No. chickens discarded from the flock	Reasons for discarding	Total chickens

B2. Breed of chickens are now raised in your flocks (if >1 breed, indicate names of all breeds):

[specify: ___________________________________]

B3. Total surfaces of areas now used to keep the chickens: [_______]m^2

B4. What type of litter/bedding currently is used for chickens:

○ Nothing ○ Rice husks ○ Straws ○ Wood shaving ○ Other, specify [________________]

B5. Do you provide heating to your chicken during brooding period?

○ No ○Yes, how long for heating [______] days

C. OTHER ANIMALS IN THE FARM

C1. Do you now keep in your farm other **chickens in addition to the target flock**?

○ No ○ Yes; How many? [__________] chicken

C2. Indicate the **age of these other chickens** (if mixed or several flocks, indicate range):

[____|-|____] ○ Days ○ Weeks ○ Months

Purpose (click all that apply): ○ Meat ○ Eggs ○ Fighting ○ Other; Specify: [___________________]

C3. Any other animal species on the farm in addition to chickens?

○ No ○ Yes (fill below numbers of each)

Quails	[______]	Ducks	[______]
Pidgeons	[______]	Muscovy ducks	[______]
Geese	[______]	Turkeys	[______]
Pigs	[______]	Cattles/buffaloes	[______]
Dogs	[______]	Cats	[______]

Other animal species (a), Specify animal species [______________] No: [______]

Other animal species (b), Specify [______________] No: [______]

C4. Is there a fish pond/s in your farm?

○ No ○ Yes (currently not used for fish production) ○ Yes (currently used for fish production)

D. CHICKEN HOUSE/PEN STYLE DESCRIPTION AND DRINKING WATER

D1. Describe the house/pen you use to keep your chicken now?

○ On stilts on solid groud ○ On stilts over pond/canal/river ○ On solid ground

**Questionnaire: MIDDLE-OF-CYCLE**			
**Study Identifier**	**Study Site**<br>**Nakhon Si Thammarat**	**Farm ID**<br>**[__|__]**	**Flock ID**<br>**[__|__]**

D2. Chicken pen/house floors are made of:

○ Concrete ○ Soil/earth ○ Metal ○ Wood ○ Metal mesh ○ Other (specify): [_______________]

D3. Drinking systems available to the chicks (select all that apply):

○ Trough ○ Bowl ○ Nipple drinkers ○ Other (specify): [_______________]

D4. Source of drinking water for the target flock (tick all that apply):

○ Municipal supply ○ Borehole/well ○ Rain water. ○ Pond ○ River/stream/canal

○ Other; specify [________________]

D5. If source of drinking water for chickens other than municipal supply, do you treat it?

○ No ○ Yes, by adding chemicals ○ Yes, by boiling ○ Other; specify [________________]

Questionnaire: MIDDLE-OF-CYCLE			
**Study Identifier**	**Study Site**<br>**Nakhon Si Thammarat**	**Farm ID**<br>**[__|__]**	**Flock ID**<br>**[__|__]**

E. DRUGS GIVEN TO THE FLOCK

Please provided all veterinary drugs provided weekly to the flocks. Each veterinary drug product is listed in 1 line. In case, the week that the flock is not given drug, please fill in the cell "number of drugs used" by 0.

Week	Number of drugs used	Drug ID	Commercial name of drug	Content of product	Unit	Amount of use	Unit	Given to all chickens of the flock (tick X in the box)	No. of specific chickens given drugs	Purpose of using drugs (Therapeutic/ Prophylatic/ Growth Promotion)
								☐		
								☐		
								☐		
								☐		
								☐		
								☐		
								☐		
								☐		
								☐		
								☐		
								☐		
								☐		
								☐		
								☐		
								☐		
								☐		
								☐		
								☐		
								☐		
								☐		
								☐		
								☐		
								☐		

								☐		
								☐		
								☐		
								☐		
								☐		
								☐		
								☐		
								☐		
								☐		

PICTURES OF TARGET FLOCK

[Farm ID] - [Flock ID] - [Millde-of-Cycle] - [Picture 01]

[Farm ID] - [Flock ID] - [Millde-of-Cycle] - [Picture 02]

[Farm ID] - [Flock ID] - [Millde-of-Cycle] - [Picture 03]

(*Examples: AB-01-M-01, AB-01-M-02, AB-01-M-03)*

PICTURES OF DRUGS (ANY DRUGS)

[AB-001-A]

[AB-001-B]

[AB-001-C]

**Questionnaire: MIDDLE-OF-CYCLE**			
**Study Identifier**	**Study Site**<br>**Nakhon Si Thammarat**	**Farm ID**<br>**[__|__]**	**Flock ID**<br>**[__|__]**

SAMPLING RECORD

Date of sample collection: [__|__]/[__|__]/[__|__] (dd/mm/yy)

Farm Visit Time:

☐ **Start-of-Cycle** (1 DOC)

☐ **Middle-of-Cycle** (around 4-6 weeks)

☐ **End-of-Cycle** (around 10 weeks)

Loại mẫu:

☐ **Cloacal swabs (10 chickens)**

[Farm ID] – [Flock ID] – [Sample ID]

(Example: from *AB-01-01* to *AB-01-10*)

Signature of sample collector

Full name
